# Near-Complete Sequence of a Highly Divergent Reovirus Genome Recovered from Callinectes sapidus

**DOI:** 10.1128/MRA.01278-20

**Published:** 2021-01-07

**Authors:** Mingli Zhao, Emily M. Flowers, Eric J. Schott

**Affiliations:** a Institute of Marine and Environmental Technology, University of Maryland, Baltimore County, Baltimore, Maryland, USA; b Institute of Marine and Environmental Technology, University of Maryland Center for Environmental Science, Baltimore, Maryland, USA; KU Leuven

## Abstract

This report describes the sequence of a reovirus genome, discovered in Callinectes sapidus in Brazil. The genome sequence of Callinectes sapidus reovirus 2 (CsRV2) consists of 12 segments that encode 13 putative proteins. The predicted RNA-dependent RNA polymerase is highly similar to that of Eriocheir sinensis reovirus 905, suggesting that CsRV2 also belongs to the genus *Cardoreovirus*.

## Abstract (precis)

This report describes the sequence of a reovirus genome, discovered in Callinectes sapidus in Brazil. The genome sequence of CsRV2 consists of 12 segments that encode 13 putative proteins. The predicted RNA-dependent RNA polymerase is highly similar to that of Eriocheir sinensis reovirus 905, suggesting that CsRV2 also belongs to the genus *Cardoreovirus*.

## ANNOUNCEMENT

The Atlantic blue crab, Callinectes sapidus, is an estuarine keystone species that functions as both predator and prey in food webs and supports a multimillion dollar fishery along the western Atlantic coast from the U.S. mid-Atlantic to southern Brazil (1, 2).

**FIG 1:**
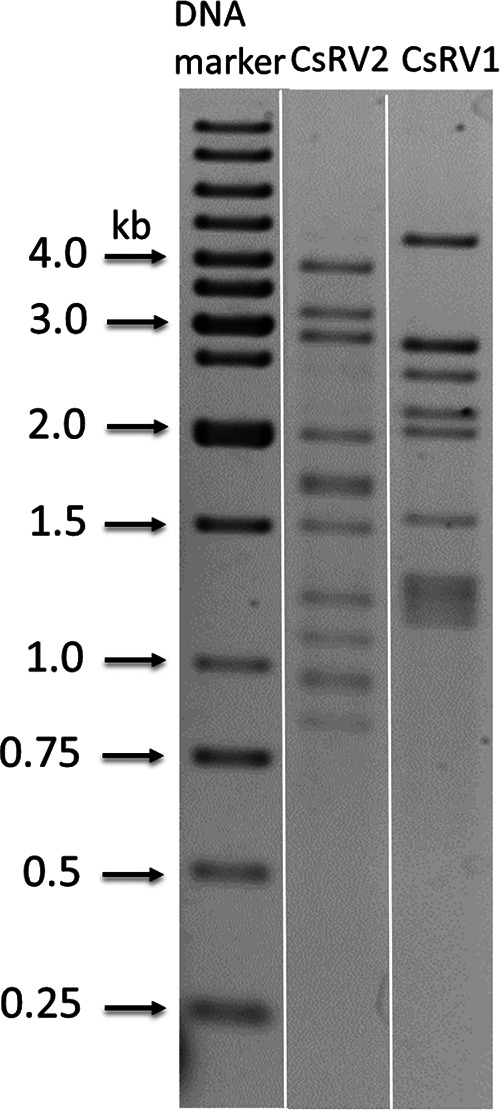
Agarose gel electrophoresis of CsRV1 and CsRV2. CsRV1 RNA was extracted from an infected *C. sapidus* specimen collected in Tramandaí, Brazil. dsRNA purified by CF11 cellulose chromatography methods as described by Bowers et al. (4) was electrophoresed in 1% agarose and visualized by ethidium bromide staining.

Reoviruses are nonenveloped icosahedral viruses with genomes comprised of 9 to 12 segments of linear double-stranded RNA (dsRNA). They have been found in diverse host species, including crabs (3). In studies on viruses of *C. sapidus* captured near Tramandaí, Brazil, we discovered a novel reovirus dsRNA that showed an electrophoretic genome organization distinct from that of *Callinectes sapidus* reovirus 1 (CsRV1) (4–9) but similar to that of *Eriocheir sinensis* reovirus 905 (EsRV905) (10), with a pattern of 3/4/5 and an estimated size of ∼21.4 kbp based on gel migration (Fig. 1). We refer to the putative reovirus represented by this dsRNA as CsRV2.

Total RNA was extracted from the muscle of an infected *C. sapidus* specimen using a phenol-guanidinium method and visualized on agarose gels (5). dsRNA was purified by CF11 cellulose chromatography as previously described (4) and used for cDNA synthesis with barcoded octamers (5′-GGCGGAGCTCTGCAGATATC-NNNNNNNN-3’) (11). The resulting cDNA was amplified by PCR using barcode primers (5′-GGCGGAGCTCTGCAGATATC-3′). PCR products of 250 to 500 bp were selected and obtained by agarose gel purification, and DNA library preparation was performed using the NEBNext Ultra DNA library prep kit following the manufacturer’s recommendations (New England BioLabs [NEB], Ipswich, MA). The library was sequenced in a 2 × 250-bp paired-end configuration on the MiSeq platform with a MiSeq reagent kit v3 (Illumina, San Diego, CA). CLC Genomics Workbench 9.5.2 (Qiagen) was used for quality trimming, removal of barcode sequences, and *de novo* assembly of the sequencing reads. The 12 viral contigs integrated 51,168 reads (67.1% of the total reads) and defined 21,109 nucleotides (nt) at 560-fold average coverage with a mean G+C content of 41.4%. The sizes of most contigs are consistent with the apparent dsRNA bands on the electrophoresis gel (Table 1 and Fig. 1), with the exception of S2 and S11, which are smaller than the dsRNA bands. The 12 contigs lack conserved 5′ or 3′ termini.

**TABLE 1:** Annotation of the CsRV2 genome sequence

CsRV2 segment	Size (nt)	Major ORF coordinates	Protein name(s)	CsRV2 GenBank accession no.	Closest sequence	Amino acid identity (%)	GenBank accession no.
1	3,742	29–3685	VP1	MW208677	EsRV905	79	Q698V5
2	3,024	25–2952	VP2	MW208678	Kadipiro virus	23	NP_694470
3	2,807	77–2476	VP3	MW208679	Liao ning virus	35	AVP49973
4	1,936	145–1851	VP4	MW208680	Liao ning virus	26	AVP72169
5	1,679	23–484	VP5A	MW208681			
		447–1583	VP5B				
6	1,631	209–1399	VP6	MW208682			
7	1,531	104–1456	VP7	MW208683			
8	1,186	23–1015	VP8	MW208684			
9	1,062	68–670	VP9	MW208685			
10	923	126–836	VP10	MW208686			
11	798	84–728	VP11	MW208687			
12	790	16–522	VP12	MW208688			

The 12 assembled segments of CsRV2 were annotated by BLASTX and BLASTN comparisons with GenBank using default parameters. A single open reading frame (ORF) was identified in each segment, with the exception that segment 5 (S5) contains two partially overlapping ORFs. S1 shows significant similarity to the putative EsRV905 *RdRp* gene, with 72% nucleotide identity and 79% amino acid identity (GenBank accession no. AY542965 and Q698V5, respectively), suggesting that CsRV2 belongs to the same genus as EsRV905, *Cardoreovirus*. Aside from the *RdRp* gene, no other sequences of EsRV905 are available in public databases. The GenBank entry with the second highest similarity (29%) to S1 of CsRV2 is the *RdRp* of the Liao ning virus, in the genus *Seadornavirus* (YP_460026) (12–14).

### Data availability.

The complete genome sequence of CsRV2 has been deposited in GenBank under accession no. MW208677 to MW208688. Raw sequencing data are registered in the NCBI SRA database under accession no. SRR13068891.

## References

[1] NOAA. 2019. NOAA landings. 2019. https://foss.nmfs.noaa.gov.

[2] Hungria DB, Tavares CPDS, Pereira LÂ, da Silva UDAT, Ostrensky A. 2017. Global status of production and commercialization of soft-shell crabs. Aquac Int 25:2213–2226. doi:10.1007/s10499-017-0183-5.

[3] Attoui H, Mertens PPC, Becnel J, Belaganahalli S, Bergoin M, Brussaard CP, Chappell JD, Ciarlet M, del Vas M, Dermody TS, Dormitzer PR, Duncan R, Fcang Q, Graham R, Guglielmi KM, Harding RM, Hillman B, Makkay A, Marzachì C, Matthijnssens J, Milne RG, Mohd Jaafar F, Mori H, Noordeloos AA, Omura T, Patton JT, Rao S, Maan M, Stoltz D, Suzuki N, Upadhyaya NM, Wei C, Zhou H. 2012. Family Reoviridae, p 541–637. *In* King AMQ, Adams MJ, Carstens EB, Lefkowitz EJ (ed), Virus taxonomy: classification and nomenclature of viruses. Ninth report of the International Committee on Taxonomy of Viruses. Academic Press, San Diego, CA.

[4] Bowers HA, Messick GA, Hanif A, Jagus R, Carrion L, Zmora O, Schott EJ. 2010. Physicochemical properties of double-stranded RNA used to discover a reo-like virus from blue crab *Callinectes sapidus*. Dis Aquat Organ 93:17–29. doi:10.3354/dao02280. 21290893

[5] Flowers EM, Simmonds K, Messick GA, Sullivan L, Schott EJ. 2016. PCR-based prevalence of a fatal reovirus of the blue crab, *Callinectes sapidus* (Rathbun) along the northern Atlantic coast of the USA. J Fish Dis 39:705–714. doi:10.1111/jfd.12403. 26249243 PMC5324600

[6] Flowers EM, Johnson AF, Aguilar R, Schott EJ. 2018. Prevalence of the pathogenic crustacean virus *Callinectes sapidus* reovirus 1 near flow-through blue crab aquaculture in Chesapeake Bay, USA. Dis Aquat Organ 129:135–144. doi:10.3354/dao03232. 29972374

[7] Flowers EM, Bachvaroff TR, Warg JV, Neill JD, Killian ML, Vinagre AS, Brown S, Almeida ASE, Schott EJ. 2016. Genome sequence analysis of CsRV1: a pathogenic reovirus that infects the blue crab Callinectes sapidus across its trans-hemispheric range. Front Microbiol 7:126. doi:10.3389/fmicb.2016.00126. 26904003 PMC4748042

[8] Spitznagel MI, Small HJ, Lively JA, Shields JD, Schott EJ. 2019. Investigating risk factors for mortality and reovirus infection in aquaculture production of soft-shell blue crabs (*Callinectes sapidus*). Aquaculture 502:289–295. doi:10.1016/j.aquaculture.2018.12.051.

[9] Zhao M, Behringer DC, Bojko J, Kough AS, Plough L, Tavares CPDS, Aguilar-Perera A, Reynoso OS, Seepersad G, Maharaj O, Sanders MB, Carnales D, Fabiano G, Carnevia D, Freeman MA, Atherley NAM, Medero-Hernández LD, Schott EJ. 2020. Climate and season are associated with prevalence and distribution of trans-hemispheric blue crab reovirus (*Callinectes sapidus* reovirus 1). Mar Ecol Prog Ser 647:123–133. doi:10.3354/meps13405.

[10] Zhang S, Shi Z, Zhang J, Bonami J-R. 2004. Purification and characterization of a new reovirus from the Chinese mitten crab, *Eriocheir sinensis*. J Fish Dis 27:687–692. doi:10.1111/j.1365-2761.2004.00587.x. 15575876

[11] Neill JD, Bayles DO, Ridpath JF. 2014. Simultaneous rapid sequencing of multiple RNA virus genomes. J Virol Methods 201:68–72. doi:10.1016/j.jviromet.2014.02.016. 24589514 PMC7119728

[12] Attoui H, Jaafar FM, Belhouchet M, Tao S, Chen B, Liang G, Tesh RB, de Micco P, de Lamballerie X. 2006. Liao ning virus, a new Chinese seadornavirus that replicates in transformed and embryonic mammalian cells. J Gen Virol 87:199–208. doi:10.1099/vir.0.81294-0. 16361432

[13] Zhang W, Li F, Liu A, Lin X, Fu S, Song J, Liu G, Shao N, Tao Z, Wang Q, He Y, Lei W, Liang G, Xu A, Zhao L, Wang H. 2018. Identification and genetic analysis of Kadipiro virus isolated in Shandong Province, China. Virol J 15:64. doi:10.1186/s12985-018-0966-y. 29625620 PMC5889548

[14] Prow NA, Mah MG, Deerain JM, Warrilow D, Colmant AMG, O'Brien CA, Harrison JJ, McLean BJ, Hewlett EK, Piyasena TBH, Hall-Mendelin S, van den Hurk AF, Watterson D, Huang B, Schulz BL, Webb CE, Johansen CA, Chow WK, Hobson-Peters J, Cazier C, Coffey LL, Faddy HM, Suhrbier A, Bielefeldt-Ohmann H, Hall RA. 2018. New genotypes of Liao ning virus (LNV) in Australia exhibit an insect-specific phenotype. J Gen Virol 99:596–609. doi:10.1099/jgv.0.001038. 29533743

